# Supplementary material for: Microbial ecology of northern Gulf of Mexico estuarine waters
Source: mSystems. 2024 Jul 9;9(8):e01318-23. doi: 10.1128/msystems.01318-23 (PMC11334486; doi:10.1128/msystems.01318-23)
Supplement: Figure S1 — Top 25 relative abundance curve. [file msystems.01318-23-s0001.pdf]

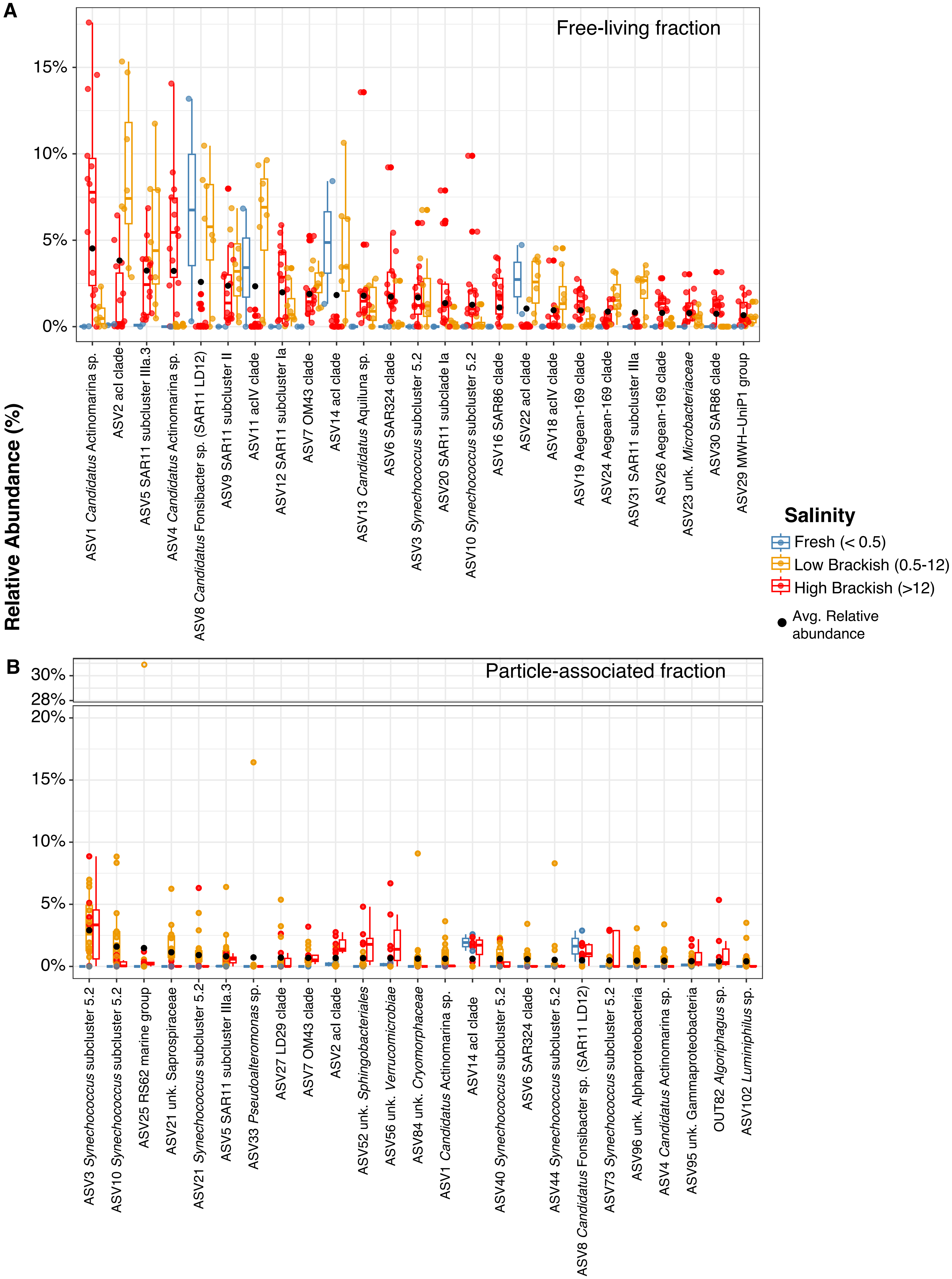

Figure S1. Rank abundances of the 25 most abundant ASVS from all sites based on the average relative abundance in the free-living fraction (A) and particle-associated fraction (B). The boxes indicate the interquartile range (IQR) of the data, with vertical lines indicating the upper and lower extremes according to 1.5 × IQR. Horizontal lines within each box indicate the median. The data points comprising the distribution are plotted on top of the boxplots. The color of the dot represents the broad salinity classification of fresh (blue, < 0.5 salinity), low salinity (orange, < 12 salinity), and high salinity (red, > 12 salinity). The black dot is the average relative abundance across all sites.
