# Supplementary material for: Microbial ecology of northern Gulf of Mexico estuarine waters
Source: mSystems. 2024 Jul 9;9(8):e01318-23. doi: 10.1128/msystems.01318-23 (PMC11334486; doi:10.1128/msystems.01318-23)
Supplement: Figure S2 — Alpha diversity of samples. [file msystems.01318-23-s0002.pdf]

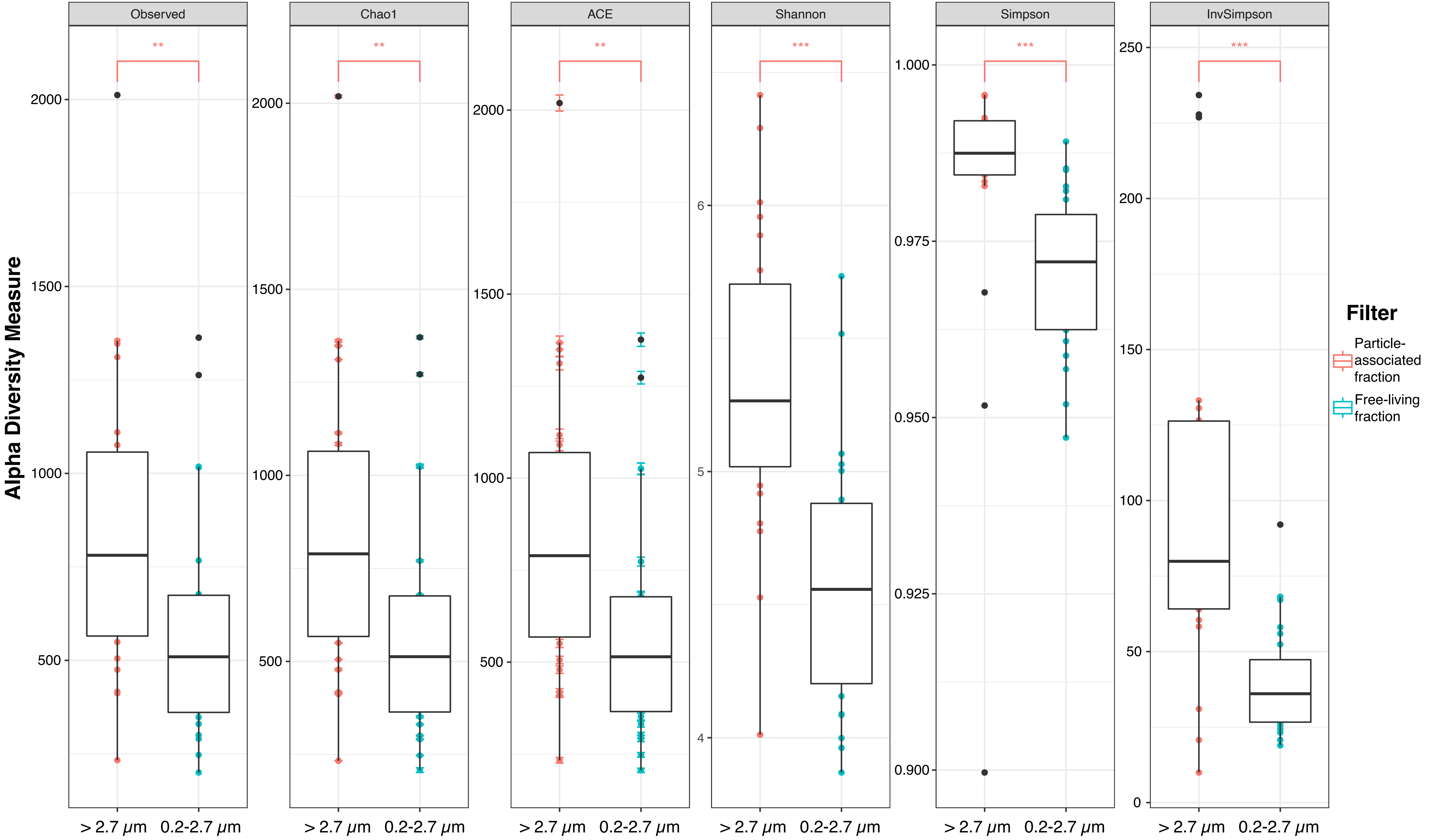

Figure S2. Alpha diversity was calculated for the free-living and particle-associated size fractions. The boxes indicate the interquartile range (IQR) of the data, with vertical lines indicating the upper and lower extremes according to 1.5 × IQR. Horizontal lines within each box indicate the median. The data points comprising the distribution are plotted on top of the boxplots. Asterisks indicate the strength of significance as calculated using a one-way ANOVA ("***"=0.001,"**"=0.01,"*"=0.05).
