# Supplementary figures and images for: Microbial ecology of northern Gulf of Mexico estuarine waters
Source: mSystems. 2024 Jul 9;9(8):e01318-23. doi: 10.1128/msystems.01318-23 (PMC11334486; doi:10.1128/msystems.01318-23)

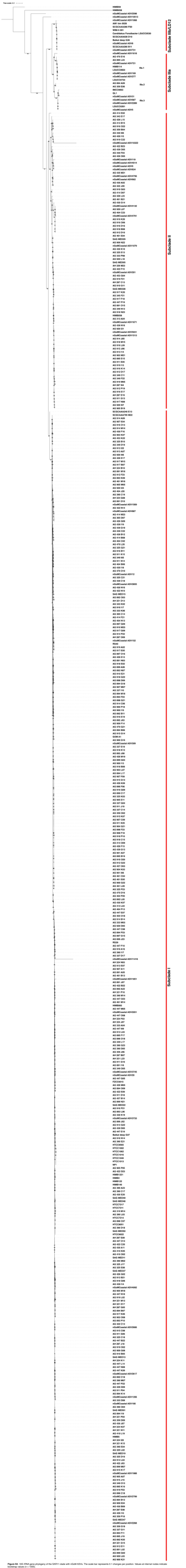

Supplement: Figure S3 — Phylogenetic tree of SAR11 with nGoM amplicons. [file msystems.01318-23-s0003.pdf]
