# Supplementary material for: Microbial ecology of northern Gulf of Mexico estuarine waters
Source: mSystems. 2024 Jul 9;9(8):e01318-23. doi: 10.1128/msystems.01318-23 (PMC11334486; doi:10.1128/msystems.01318-23)
Supplement: Figure S5 — Frequency plot of rho values (Spearman rank correlation) from salinity analysis. [file msystems.01318-23-s0005.pdf]

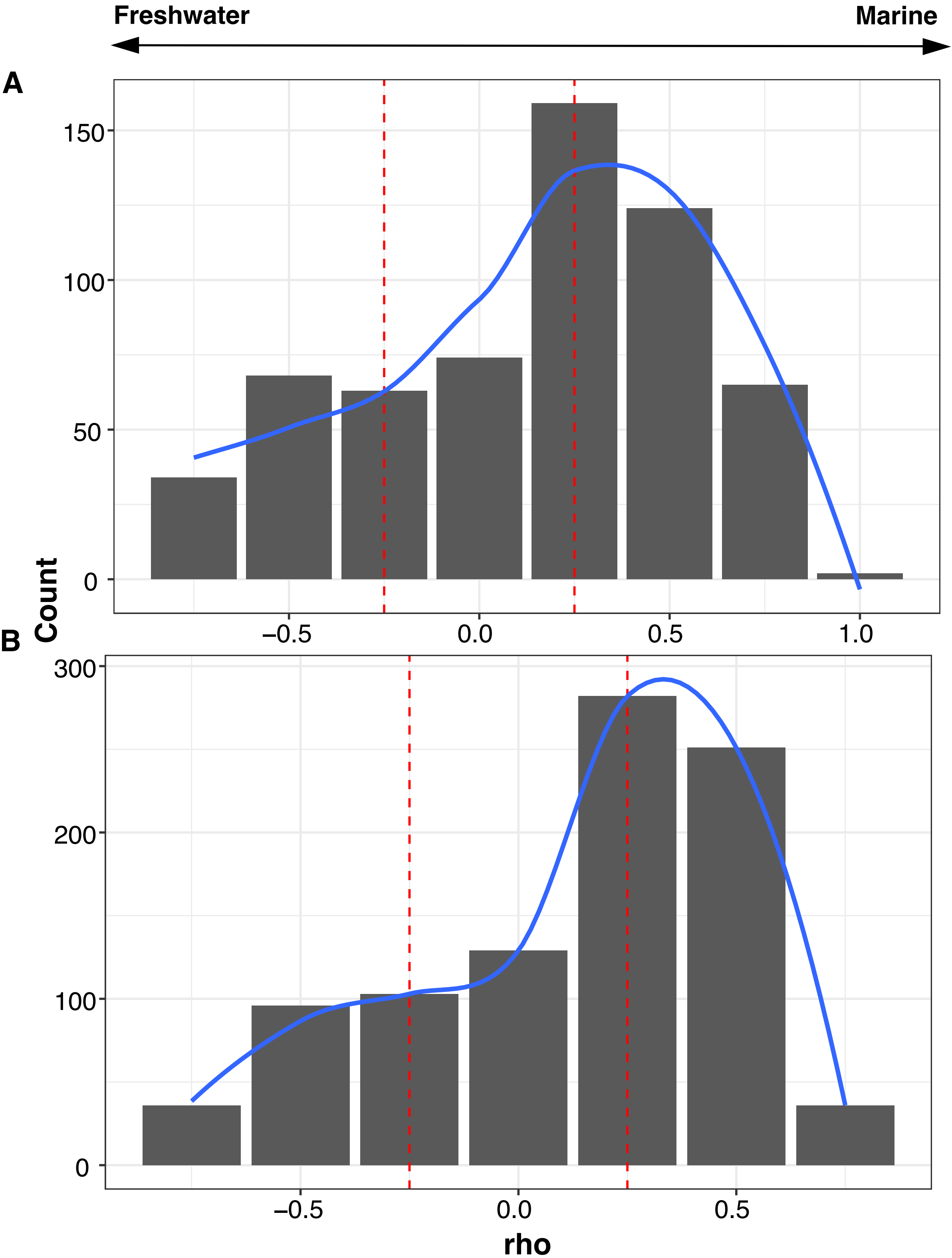

Figure S5. The relative frequency of ASVs by the observed two-sided Spearman's rank correlation coefficient (rho) from the free-living (A) and particle-associated (B) fraction communities. Two-sided rho values were rounded to the nearest quarter decimal. Between the red dashed lines are taxa poorly correlated (-0.25 to 0.25) to salinity. A blue nonlinear regression line is provided as a visual aid for trends.
