# Supplementary material for: Microbial ecology of northern Gulf of Mexico estuarine waters
Source: mSystems. 2024 Jul 9;9(8):e01318-23. doi: 10.1128/msystems.01318-23 (PMC11334486; doi:10.1128/msystems.01318-23)
Supplement: Figure S4 — Phylogenetic tree of acI clade with nGoM amplicons. [file msystems.01318-23-s0004.pdf]

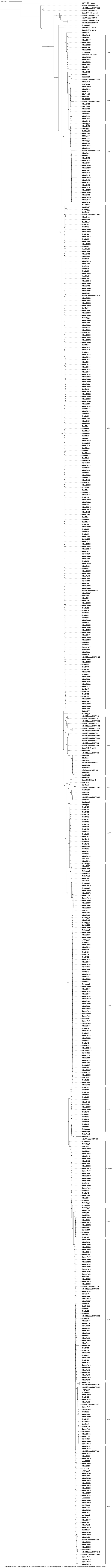

Figure S4. 16S rRNA gene phylogeny of the acI clade with nGoM ASVs. The scale bar represents 0.1 changes per position. Values at internal nodes indicate bootstrap values (n = 1000).
